# The association between vitamin D and the progression of diabetic nephropathy: insights into potential mechanisms

**DOI:** 10.3389/fmed.2024.1388074

**Published:** 2024-06-24

**Authors:** Jiachen Gao, Xiujun Song, Hongling Ou, Xiyu Cheng, Lishu Zhang, Chen Liu, Ya Dong, Xinru Wang

**Affiliations:** ^1^The PLA Rocket Force Characteristic Medical Center, The Postgraduate Training Base of Jinzhou Medical University, Beijing, China; ^2^Department of Clinical Laboratory, The PLA Rocket Force Characteristic Medical Center, Beijing, China; ^3^College of Life Sciences and Bioengineering, School of Physical Science and Engineering, Beijing Jiaotong University, Beijing, China

**Keywords:** type 2 diabetes, diabetic nephropathy, vitamin D, 25-OH-D3, bioinformatics

## Abstract

**Aims:**

Vitamin D deficiency (VDD) is prevalent in the population, with inadequate intake, impaired absorption and metabolism as the main causative factors. VDD increases the risk of developing chronic diseases such as type 2 diabetes mellitus (T2DM) and diabetic nephropathy (DN), but the molecular mechanisms underlying this phenomenon are not known. The aim of this study was to investigate the association and potential mechanisms of vitamin D levels with the progression of DN by analyzing general clinical data and using bioinformatics methods.

**Methods:**

The study included 567 diabetes mellitus type 2 (T2DM) patients from the Rocket Force Characteristic Medical Center as the case group and 221 healthy examinees as the normal control group. T2DM patients were categorized into T2DM, early diabetic nephropathy (EDN), and advanced diabetic nephropathy (ADN) based on the progression of diabetic nephropathy. The renal RNA-seq and scRNA-seq data of patients with DN were mined from public databases, and the differential expression of vitamin D-related genes in normal-EDN-ADN was analyzed by bioinformatics method, protein interaction network was constructed, immune infiltration was evaluated, single cell map was drawn, and potential mechanisms of VD and DN interaction were explored.

**Results:**

Chi-square test showed that vitamin D level was significantly negatively correlated with DN progression (*p* < 0.001). Bioinformatics showed that the expression of vitamin D-related cytochrome P450 family genes was down-regulated, and TLR4 and other related inflammatory genes were abnormally up-regulated with the progression of DN. Vitamin D metabolism disturbance up-regulate “Nf-Kappa B signaling pathway,” B cell receptor signaling pathway and other immune regulation and insulin resistance related pathways, and inhibit a variety of metabolic pathways. In addition, vitamin D metabolism disturbance are strongly associated with the development of diabetic cardiomyopathy and several neurological disease complications.

**Conclusion:**

VDD or vitamin D metabolism disturbance is positively associated with the severity of renal injury. The mechanisms may involve abnormal regulation of the immune system by vitamin D metabolism disturbance, metabolic suppression, upregulation of insulin resistance and inflammatory signalling pathways.

## 1 Introduction

Diabetes is a complex multifactorial metabolic syndrome characterized by hyperglycemia resulting from insufficient insulin secretion or insulin resistance, with 90% of diabetic patients diagnosed with diabetes mellitus type 2 (T2DM) (1, 2). Diabetic nephropathy (DN) is a chronic kidney damage caused by diabetes, occurring in approximately 40% of T2DM patients, characterized by increased proteinuria, decreased glomerular filtration rate (GFR), and renal failure (3). It has been reported that the number of DN patients will increase with the rising prevalence of diabetes globally, and the progression of DN can lead to severe infections and cardiovascular diseases (4). Therefore, there is an urgent need to clarify the pathogenic mechanisms of DN, identify more targeted treatment methods, and delay or prevent the deterioration of renal function.

Vitamin D is an essential nutritional supplement, with 25-OH-D3 being the primary form of vitamin D in the body. Clinical assessment of vitamin D levels is often done by measuring the concentration of 25-OH-D3 in serum. However, vitamin D deficiency (VDD) is widespread in the population, primarily due to inadequate intake, absorption, and metabolic disturbance. Meta-analyses have shown that supplementing vitamin D for individuals at risk of diabetes can reduce the risk of developing T2DM (5). Additionally, vitamin D can regulate the renin-angiotensin-aldosterone system (RAAS) to protect the kidneys, indicating potential applications in the prevention and treatment of DN (6). Other studies have suggested an association between high doses of vitamin D and improved progression in DN patients with type 1 diabetes (T1DM) (7). Low vitamin D levels have been correlated with the progression of DN and a decline in estimated glomerular filtration rate (eGFR) in DN patients (8). However, whether increasing vitamin D levels can reduce the risk of DN in diabetic patients and the underlying mechanisms remain unclear. There is currently no consensus on the predictive and therapeutic potential of vitamin D levels in the progression of DN. This study aims to explore the correlation between VDD and the progression of DN, combining single-cell RNA sequencing and bulk RNA-seq analyses to uncover the potential mechanisms through which VDD influences the progression of DN, providing new experimental evidence for the prevention and treatment of DN.

## 2 Materials and methods

### 2.1 Study subjects

This study included 567 patients diagnosed with T2DM and DN who sought medical care at PLA Rocket Force Characteristic Medical Center from January 2019 to January 2023. Additionally, 221 healthy individuals undergoing routine health examinations were enrolled as the control group. The inclusion criteria for cases were based on the diagnostic standards for diabetes care set forth by the American Diabetes Association (ADA) (9).

Diagnosis of DN: DN-diagnosed patients met the following criteria (10). Urinary albumin/creatinine ratio (UACR) ≥30 mg/g in at least 2 measurements over a period of 3 to 6 months. eGFR <60 mL/min/1.73 m^2^ sustained for more than 3 months. Renal biopsy pathology changes consistent with DN.

Exclusion criteria: Systolic blood pressure ≥140 mmHg, diastolic blood pressure ≥90 mmHg. Age ≤30 years or ≥90 years. BMI ≥35 or ≤18.5 kg/m^2^. Diabetes induced by corticosteroids. Gestational diabetes. Chronic kidney disease caused by other aetiology. Malignant tumors. Infectious diseases. Thyroid dysfunction. Acute or chronic inflammation. Patients who had taken vitamin D or calcium supplements within 6 months.

T2DM patients were categorized based on at least two random measurements of urine albumin creatinine ratio (UACR) and continuous monitoring of estimated glomerular filtration rate (eGFR) for over 3 months. According to the Chinese Guidelines for the Prevention and Treatment of Diabetic Kidney Disease, diabetes patients were classified into T2DM, EDN, and ADN groups based on the risk of DN progression, T2DM group was defined as UACR <30 (mg/g) and eGFR ≥60 (mL/min/1.73m^2^), EDN as 30 ≤UACR <300 (mg/g) or 45 ≤eGFR <60, and ADN as UACR >300 (mg/g) or eGFR <45 (mL/min/1.73m^2^) (10). We measured total combined 25-OH-D3 in the serum. Following the clinical practice guidelines of the Endocrine Society, vitamin D levels were categorized as extreme deficiency (vitamin D <10 ng/mL), deficiency (10 ≤ vitamin D < 20 ng/mL), insufficient (20 ≤ vitamin D < 30 ng/mL), and normal (vitamin D > 30 ng/mL) (11).

### 2.2 Experimental methods and instruments

Upon admission, professional healthcare personnel measured and calculated baseline physiological indicators, including age, sex, blood pressure (BP), and body mass index (BMI).

Biochemical and immune assays were conducted as follows: fasting blood glucose (GLU) was measured using the glucose oxidase method. glycated hemoglobin (HbA1c) levels were determined through high-performance liquid chromatography. Prothrombin time (PT) was assessed using coagulation methods. Total triiodothyronine (TT3) levels were measured by competitive assay. Thyroid-stimulating hormone (TSH), parathyroid hormone (PTH), and bone gla protein (BGP) were analyzed using the sandwich immunoassay method. C-peptide (C-P) was assayed using sandwich immunoassay. Blood calcium (Ca) was determined using the phenolphthalein complex copper method. Blood uric acid (UA), blood creatinine (Scr), high-density lipoprotein (HDL), low-density lipoprotein (LDL), triglycerides (TG), and total cholesterol (CHOL) were measured using homogeneous enzymatic colorimetry. Serum 25-OH-D3 concentration was assayed using electrochemiluminescence immunoanalysis. Random urine albumin/creatinine ratio (UACR) was determined using immunoturbidimetry. All biochemical and immune assay reagent kits were sourced from Roche and were tested on the Roche Cobas 8000 fully automated biochemical and immune flow line. The estimated glomerular filtration rate (eGFR) was calculated using the modified MDRD equation:


$$\begin{array}{l} f\left(\mathrm{eGFR}\right)=175*Scr\;{\left(\mathrm{mg}/\mathrm{dL}\right)}^{-1.234}*{\left(\mathrm{years}\right)}^{-0.179}*\\ \left(0.79\;for\;\mathrm{female}\;\mathrm{patients}\right) \end{array}$$

### 2.3 Vitamin D-related gene sets and sequencing data acquisition

The vitamin D-related gene set was assembled by consolidating information from the Comparative Toxicogenomics Database (CTD) and Genecards. For gene expression data, two datasets were sourced from the Gene Expression Omnibus (GEO) database.^1^ GSE142025: this dataset encompasses gene expression microarray data from 21 ADN, 6 EDN, and 9 normal samples. GSE111154: This dataset provides gene expression microarray data from 4 EDN samples. scRNA-seq data were obtained from the GSE195460 dataset, including scRNA-seq data from 5 patients with DN. The sample data in the open database above were obtained from kidney tissue biopsies.

### 2.4 Selection and temporal trend analysis of vitamin D DEGs

The combat function from the sva package (version 3.46.0) was employed to mitigate batch effects between the two gene expression array datasets. Visualization of the data was carried out using the scatterplot3d package (version 0.3–43) for principal component analysis (PCA). Differential expression analysis was performed using limma (version 3.54.2) between the EDN group and the normal group, as well as between the ADN group and the EDN group (*p*-value <0.05, |log2FC| > 1) (12). The resulting set of differentially expressed vitamin D-related genes was obtained. The stages of normal-EDN-ADN were delineated, and the Mfuzz package was utilized to analyze the gene expression trends of vitamin D DEGs across these three stages. Visualization and analysis of the clusters were conducted using ClusterGVis (version 0.0.9). Enrichment analysis was performed on the clustered genes, and enriched pathways were examined.

### 2.5 Patient stratification and differential enrichment analysis in DN patients

The expression matrix of vitamin D DEGs in ADN patients underwent dimensionality reduction using the Uniform Manifold Approximation and Projection (UMAP) algorithm. Subsequently, sample clustering was performed using the *k*-means algorithm. The number of clusters is set to 2, and samples with similar expression patterns will be clustered together, while those with dissimilar expression patterns will be separated. Finally, cluster 1 and cluster 2 have different expression patterns of vitamin D related DEGs. Limma differential analysis was conducted between the two groups (*p*-value <0.05, |log2FC| > 1). Gene set enrichment analysis (GSEA) was employed to explore activated and inhibited pathways related to the DGEs. Additionally, the gene set variation analysis (GSVA) algorithm (version 1.46.0) was utilized to calculate pathway scores within different patient subgroups.

### 2.6 Construction and visualization of PPI network

The protein–protein interaction network (PPI) was constructed using the STRING database.^2^ The MCODE plugin was applied to identify core subnetworks within the PPI network, with specific parameters set as follows: degree cut-off = 2, node density cut-off = 0.1, node score cut-off = 0.2, *k*-core = 2, and maximum depth = 100. The hub genes in the PPI network were identified using the MCC (Maximal Clique Centrality) algorithm within the CytoHubba plugin. Cytoscape was employed for visualizing the network, and Metascape^3^ was utilized for enrichment analysis.

### 2.7 Immune infiltration analysis

The ESTIMATE algorithm (13) was employed to calculate the overall immune score for the two groups of DN patients. Subsequently, the ssGSEA algorithm (14) was utilized for infiltration analysis of 28 immune-related cell types, providing insights into the immune infiltration status of various cellular subgroups.

### 2.8 Construction of scRNA-seq Atlas

The Doubletfinder algorithm (15) was applied for doublet removal, and quality control (QC) was conducted with specific conditions (nFeature RNA >300 & nFeature RNA <4,000 & mt percent <10 & HB percent <3). Subsequently, the harmony algorithm (16) was utilized for the integration of scRNA-seq samples. The Seurat package (17) was then used for dimensionality reduction, clustering, and subsequent single-cell analysis. Relevant markers were obtained from the Cellmaker2.0 database (18) to assign names to the clustered subpopulations. The Scissor algorithm (19) was employed to integrate bulk RNA-seq and vitamin D phenotype with scRNA-seq data. The Findmarker function was used for differential analysis of Scissor cells, followed by enrichment analysis.

### 2.9 Statistical methods

Statistical analyses were performed using functions in R software (version 4.2.3). For normally distributed data, the student’s *t*-test and ANOVA were employed. In the case of non-normally distributed data, the Wilcoxon rank-sum test and Kruskal–Wallis test were applied. Pearson correlation coefficient was utilized for normally distributed data, while Spearman correlation coefficient was used for non-normally distributed data. A chi-square test was performed for the correlation of vitamin D levels between different periods of T2DM and DN. A significance level of *p* < 0.05 was considered statistically significant.

## 3 Results

### 3.1 Analysis of general clinical data

A total of 567 T2DM patients were assessed, comprising 277 males and 290 females. With the progression of diabetic nephropathy, age, uric acid (UA), serum creatinine (Scr), UACR, C-P, and glycated hemoglobin (HbA1c) in the EDN and ADN groups were significantly higher than those in the T2DM group (*p* < 0.05). Conversely, vitamin D levels and eGFR were significantly lower in the EDN and ADN groups compared to the T2DM group (*p* < 0.001). There were no statistically significant differences in indicators such as GLU, PT, TT3, TSH, BGP, Ca^2+^, HDL, LDL, TG, CHOL, BP, etc., among the T2DM-EDN-ADN three groups (*p* > 0.05) (Table 1).

**Table 1:** Analysis of clinical indicators in different classifications of patients.

Clinical indicator	Normal (221)	T2DM (*N* = 379)	EDN (*N* = 148)	ADN (*N* = 40)	*F*/*χ*^2^ value	*p*-value
Sex (male/female)	110/111	191/188	64/84	22/18	18.173	0.41
Age (years)	59.57 ± 14.13	58.5 ± 12.7	61.8 ± 11.6	67.5 ± 11.4	11.91	<0.05
SBP (mmHg)	124 ± 14.1	122.33 ± 12.6	122.18 ± 11.2	126.57 ± 11.6	1.888	0.152
DBP (mmHg)	74.83 ± 11.21	70.97 ± 10.27	72.35 ± 9.07	70.19 ± 9.85	1.014	0.363
BMI (kg/m^2^)	22.25 ± 2.97	25.4 ± 3.33	25.6 ± 4.04	25.3 ± 4.57	0.157	0.854
LDL (mmol/L)	2.38 ± 0.99	2.7 ± 0.95	2.66 ± 0.91	2.64 ± 1.27	0.12	0.886
HDL (mmol/L)	1.24 ± 0.51	1.09 ± 0.32	1.12 ± 0.32	1.05 ± 0.31	0.915	0.401
TG (mmol/L)	2.35 (1.96, 2.62)	1.5 (1.06, 2.03)	1.37 (0.98, 2.06)	1.55 (1.17, 1.93)	0.11	0.9
CHOL (mmol/L)	4.19 ± 1.05	4.22 ± 1.22	4.22 ± 1.13	4.23 ± 1.49	0.033	0.967
UA (umol/L)	235.5 ± 79.23	315.1 ± 87.23	312.6 ± 85.22	375.4 ± 92.81	9.093	<0.05
ALB (g/L)	42.8 ± 6.5	42.1 ± 4.16	41.4 ± 4.04	37.3 ± 5.28	24.555	<0.001
Scr (mg/dL)	0.63 ± 0.19	0.75 ± 0.16	0.74 ± 0.22	1.21 ± 0.53	82.507	<0.001
TT3 (ng/mL)	0.98 ± 0.35	1.04 ± 0.25	1.02 ± 0.386	0.92 ± 0.32	2.677	0.07
PTH (pg/mL)	33.98 ± 13.13	33.92 ± 11.63	35.45 ± 11.22	34.13 ± 13.76	1.222	0.295
TSH (mU/L)	1.21 (1.08, 1.32)	1.9 (1.31, 2.72)	1.85 (1.19, 2.72)	2.21 (1.23, 3.34)	1.44	0.237
CA (mmol/L)	2.29 ± 0.17	2.35 ± 0.13	2.24 ± 0.12	2.19 ± 0.11	0.3042	0.738
PT (sec)	11 ± 1.41	10.6 ± 0.93	10.6 ± 0.83	10.3 ± 0.64	2.265	0.104
C-P (ng/mL)	2.06 (1.51, 2.59)	2.14 (1.32, 2.83)	1.88 (1.41, 2.84)	2.46 (1.65, 3.72)	3.101	<0.05
BGP (ng/mL)	7.64 (6.43, 8.61)	11.23 (9.01, 14.2)	11.24 (8.36, 14.89)	11 (8.66, 15.14)	2.6	0.075
GLU (mmol/L)	4.98 (4.72, 5.36)	7.5 (6.43, 9.71)	9.20 ± 4.73	9.69 ± 4.05	0.542	0.581
HbA1c (%)	5.1 ± 1.2	8.44 ± 2.13	9.17 ± 2.28	9.84 ± 2.43	11.35	<0.05
25-OH-D3 (ng/mL)	30.88 ± 7.67	14.3 ± 7.31	11.0 ± 6.33	6.99 ± 3.19	27.807	<0.001
eGFR (mL/min/1.73m^2^)	104.71 (101.21, 108.34)	116.58 (98.19, 133.10)	113.48 (93.85, 139.45)	59.6 (45.09, 106.9)	34.569	<0.001
UACR (mg/mmol)	4.84 (3.49, 6.22)	9.76 (5.94, 15.5)	62.35 (40.95, 97.43)	367.1 (138.8, 681.63)	24.772	<0.001

Statistical analysis was performed among T2DM-EDN-ADN patients. *χ*^2^-test was used for sex and *F*-test was used for other indicators. Normal distribution data were expressed as mean ± SD, and skewed data were expressed as median (quartile 1 and quartile 3).

**Figure 1:**
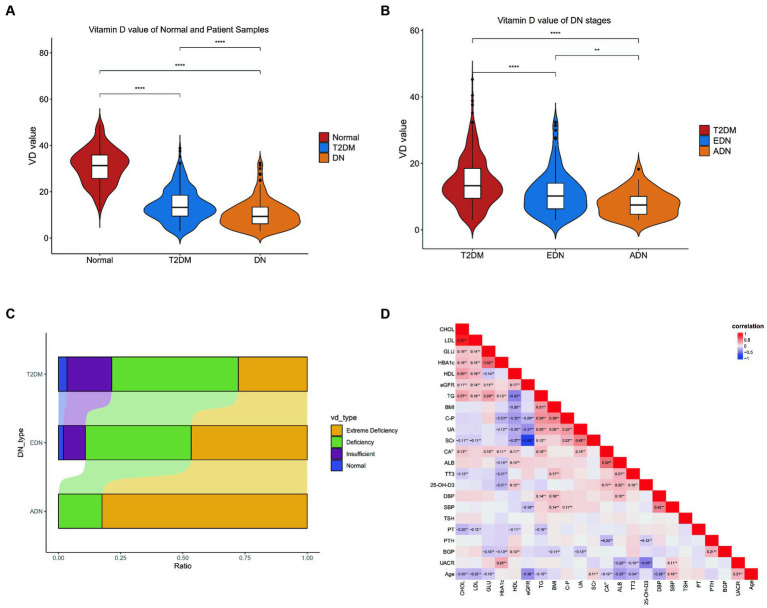
Clinical data analysis. **(A)** Differences in vitamin D levels among clinic normal, T2DM, and DN patients. **(B)** Differences in vitamin D levels between clinic T2DM and DN patients at different stages from **(A)**. The Wilcox test was used to analyze inter group differences. **(C)** Differences in VD typing between patients with DM and different stages of DN. The horizontal axis represents the proportion of patients with various VD subtypes, and the vertical axis represents the type of patients. The chi square test is used to analyze differences in grouped variables. **(D)** Correlation analysis of all clinical indicators of patients. The Spearman correlation coefficient is used to analyze correlation. *p* < 0.05 is considered statistically significant. ^ns^*p* > 0.05, ^*^p < 0.05, ^**^p < 0.01, ^***^p < 0.001, and ^****^p < 0.0001. T2DM, diabetes mellitus type 2; DN, diabetic nephropathy; EDN, early diabetic nephropathy; ADN, advanced diabetic nephropathy.

Analysis of vitamin D levels among different groups revealed that the normal group had significantly higher vitamin D levels compared to both the T2DM and DN groups. Additionally, the ADN group exhibited lower vitamin D levels compared to the T2DM and EDN groups (Figures 1A,B). Furthermore, as kidney disease progressed, the proportion of severely vitamin D-deficient patients in the ADN group increased compared to the T2DM and EDN groups. Chi-square analysis indicated a significant statistical difference (X-squared = 61.885, df = 12, *p*-value = 1.021 × 10^−8^) (Figure 1C). Correlation analysis of various clinical indicators revealed a significant negative correlation between vitamin D levels and UACR (*r* = −0.45) (Figure 1D). However, no significant correlation was observed between vitamin D levels and estimated glomerular filtration rate (eGFR).

Analysis of vitamin D levels among different groups revealed that the normal group had significantly higher vitamin D levels compared to both the T2DM and DN groups. Additionally, the ADN group exhibited lower vitamin D levels compared to the T2DM and EDN groups (Figures 1A,B). Furthermore, as kidney disease progressed, the proportion of severely vitamin D-deficient patients in the ADN group increased compared to the T2DM and EDN groups. Chi-square analysis indicated a significant statistical difference (X-squared = 61.885, df = 12, *p*-value = 1.021 × 10^−8^) (Figure 1C). Correlation analysis of various clinical indicators revealed a significant negative correlation between vitamin D levels and UACR (*r* = −0.45) (Figure 1D). However, no significant correlation was observed between vitamin D levels and estimated glomerular filtration rate (eGFR).

### 3.2 Selection of 209 vitamin D DEGs

**Figure 2:**
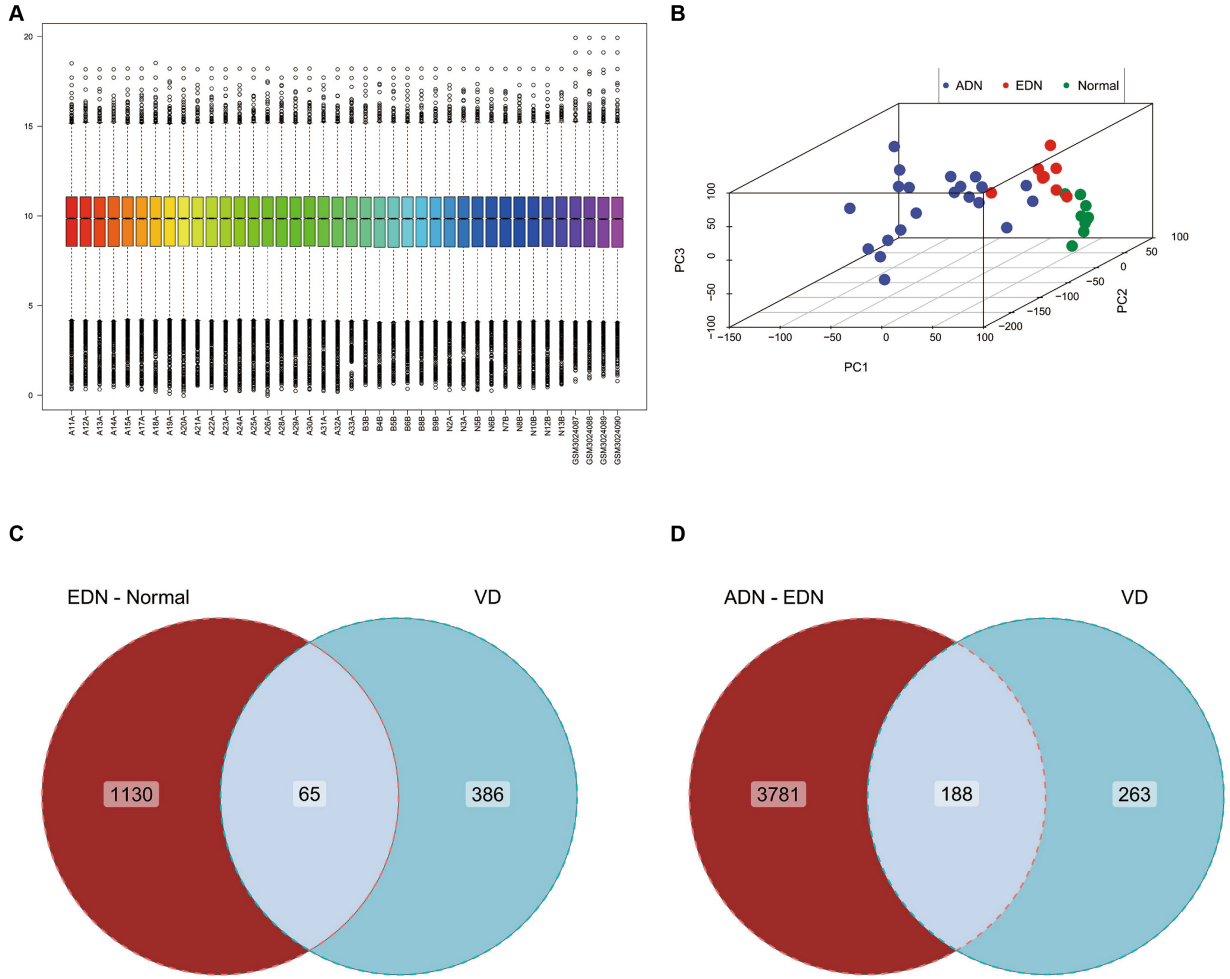
Gene expression chip data analysis of normal, EDN, and ADN patients. **(A)** Box plot of overall gene expression data for each sample after batch correction. All samples starting with A, B, and N are from GSE142025, while samples starting with GSM are from GSE111154. **(B)** 3D PCA analysis of gene expression levels in all samples. Different colors represent different sample types. **(C)** Venn diagram of the intersection of VD related genes and differentially expressed genes between EDN patient samples and normal samples. **(D)** Venn diagram of the intersection of VD related genes and differentially expressed genes between ADN patient samples and EDN patient samples. VD, VD related genes; EDN-normal, differentially expressed genes between EDN patient samples and normal samples; ADN-EDN, differentially expressed genes between ADN patient samples and EDN patient samples.

With an interaction count cutoff set at 2, 1626 vitamin D-related genes were obtained from the comparative CTD. Setting the relevance score cutoff at 5, 1,812 vitamin D-related genes were obtained from Genecards. Venn analysis of both databases identified 451 vitamin D-related genes, including VDR and CYP27B1, which were collectively termed the vitamin D-related gene set. After integrating and batch effect removal of RNA-seq data from GSE142025 and GSE111154, PCA was performed and visualized using the scatterplot3d package (Figures 2A,B). The intersection of the DEGs between EDN and normal (|log2FC| > 1, *p*-value <0.05) with the vitamin D-related gene set yielded 65 differentially expressed vitamin D-related genes. Additionally, the ADN-EDN comparison resulted in 188 differentially expressed vitamin D-related genes (|log2FC| > 1, *p*-value < 0.05). The union of these two sets finally yielded 209 vitamin D-related DEGs (Figures 2C,D).

### 3.3 Downregulation of cytochrome P450 family genes and upregulation of inflammation-related genes in vitamin D DEGs

**Figure 3:**
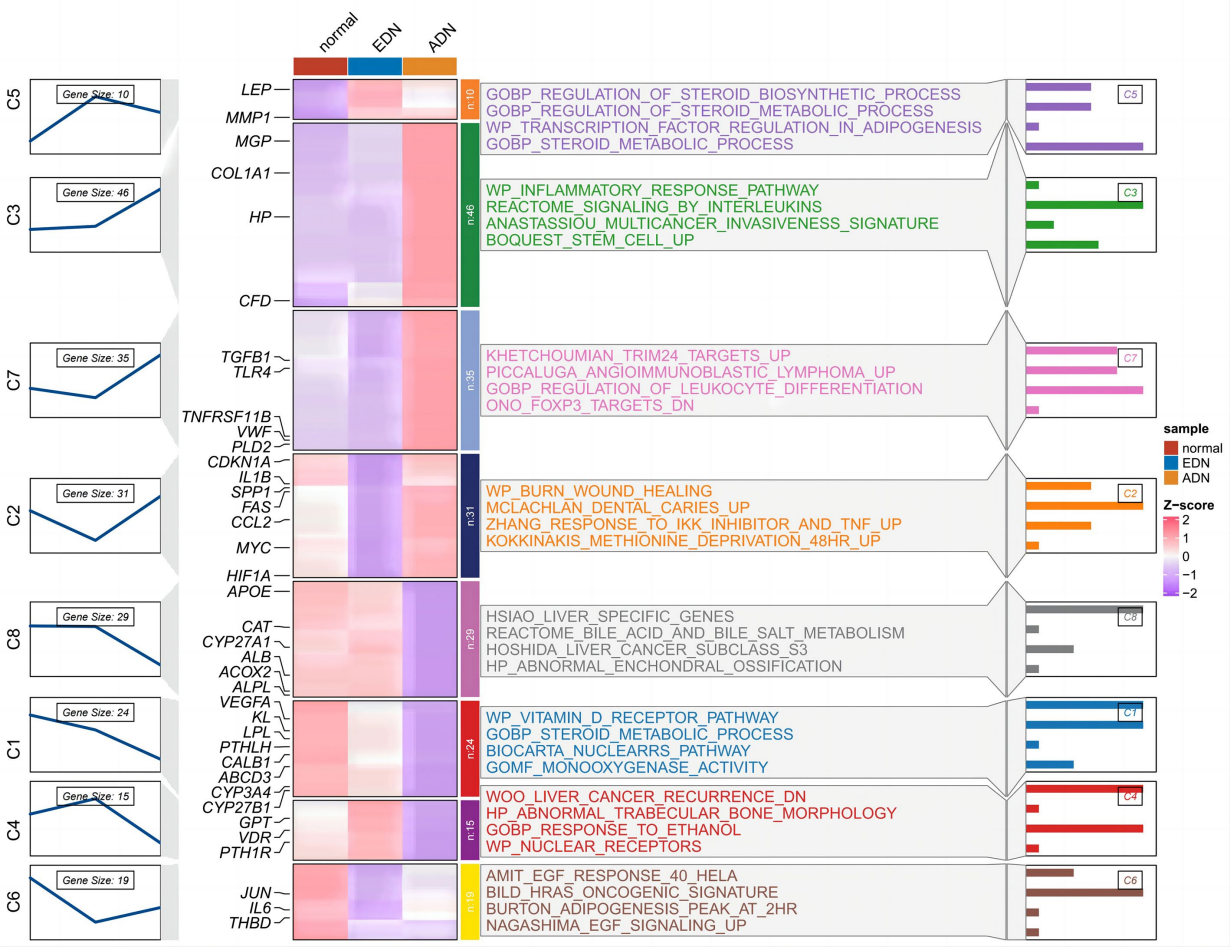
Time trend analysis of vitamin D-related DEGs in Figure 2 normal, EDN, and ADN samples. The left part shows the expression clustering of vitamin D-related DEGs, showing 8 gene expression trend clusters. In the middle are the expression status of genes and the enrichment pathways of genes of each cluster. The right section shows the −log10 (*p*-value) of each pathway.

The expression trends of vitamin D DEGs were analyzed using Mfuzz across the normal-EDN-ADN stages, as shown in Figure 3. Vitamin D DEGs were clustered into 8 groups, with some genes annotated on the heatmap. A significant continuous downregulation (cluster C1) was observed in a subset of vitamin D-related cytochrome P450 family genes, such as *CYP27A1*, *CYP27B1*, and *CYP3A4*. Enrichment analysis revealed that these genes inhibit the vitamin D receptor and oxidative metabolism pathways. Furthermore, certain vitamin D DEGs associated with inflammation, such as TLR4 and TNFRSF11B, showed a significant upregulation with the progression of kidney disease. This suggests that changes in vitamin D levels in DN patients may influence both metabolic and inflammatory pathways.

### 3.4 Upregulation of immune-related and insulin resistance-related pathways with inhibition of glucose metabolism in vitamin D metabolic disturbance

**Figure 4:**
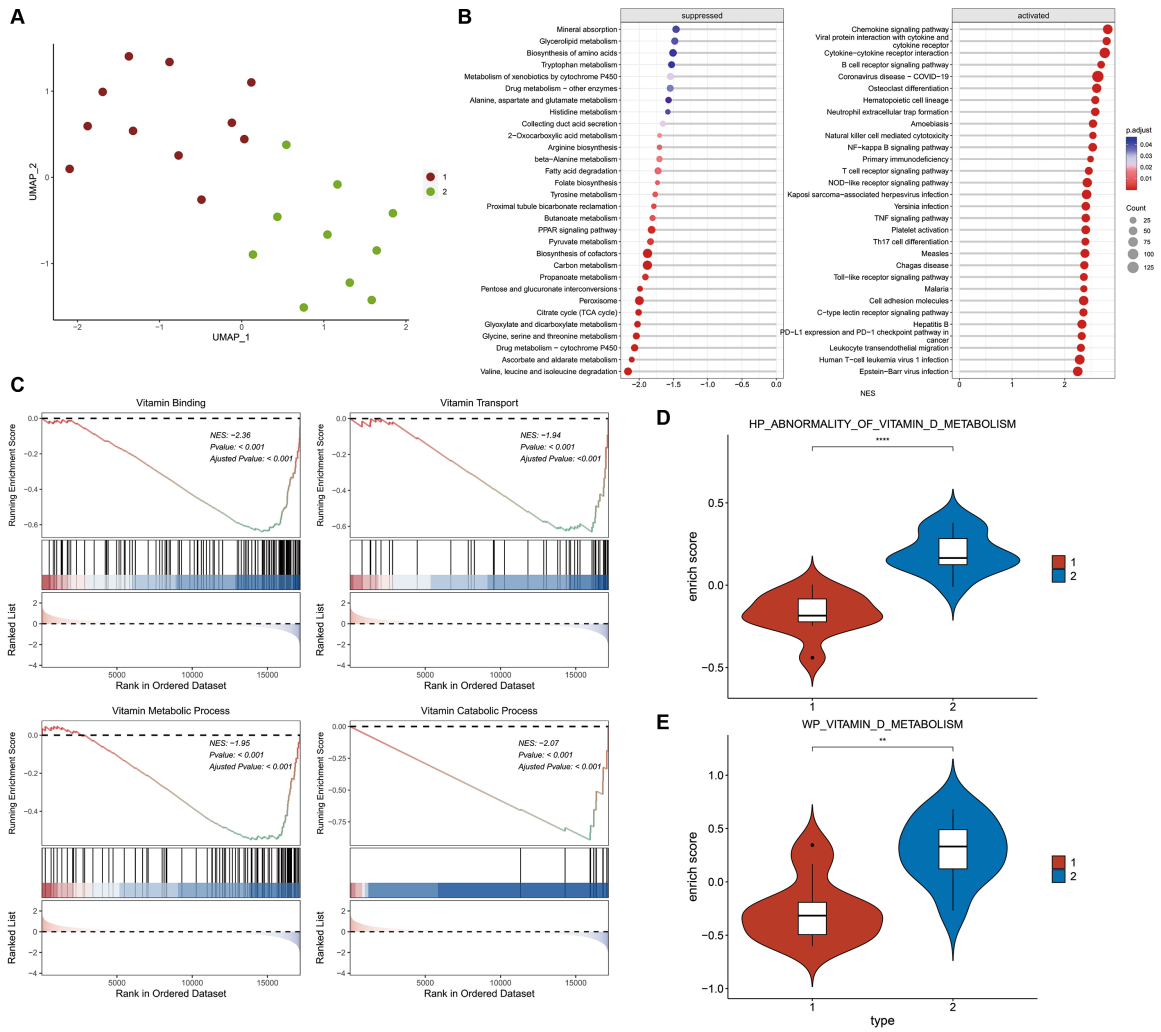
Differential and enrichment analysis of ADN patients. **(A)** Visualization results after using UMAP dimensionality reduction and *k*-means clustering. Use the vitamin D-related DEGs expression matrix of 21 ADN patients in Figure 2. The sample is divided into two groups: cluster 1 and cluster 2. Cluster 1 and cluster 2 have different expression patterns of vitamin D-related DEGs. **(B)** KEGG pathways enriched in GSEA. The horizontal axis NES represents enrichment score, positive numbers represent pathway activation, and negative numbers represent pathway suppression. **(C)** Vitamin D related pathways inhibited by cluster 1 compared to cluster 2. The first part is the line graph of gene enrichment score. The horizontal axis represents each gene in the gene set, and the vertical axis represents the corresponding running enrichment score. The peak value of the line graph is the enrichment score, which is the NES value of the gene set. The genes before the peak value are the core genes in the gene set. The middle part of the second part marks the genes located under the gene set. The third part is a distribution map of rank values for all genes, with the vertical axis reflecting the relative expression levels of each gene in the gene set. **(D,E)** GSVA scores for pathways WP vitamin D metabolism and HP abnormality of vitamin D metabolis in two groups. The Wilcox test was used to analyze inter group differences. *p* < 0.05 is considered statistically significant. ^ns^*p* > 0.05, ^*^p < 0.05, ^**^p < 0.01, ^***^p < 0.001, and ^****^p < 0.0001.

The expression matrix of vitamin D related DEGs in the kidney of ADN patients underwent dimensionality reduction and then sample clustering was performed. Twenty-one ADN patients (Figure 4A) were classified into two distinct clusters. Limma differential analysis on these clusters identified 296 upregulated genes and 473 downregulated genes (|log2FC| > 1 and *p*-value < 0.05) (Supplementary Figures S1A,B). KEGG enrichment analysis through GSEA revealed the inhibition of vitamin binding pathways in cluster 1 (Figures 4B,C). Using the GSVA algorithm, scores for two vitamin D metabolic pathways—WP VITAMIN D METABOLISM and HP ABNORMALITY OF VITAMIN D METABOLISM—were significantly lower in cluster 1, indicating a relatively severe vitamin D metabolic disturbance (Figures 4D,E, *p* < 0.001).

**Figure 5:**
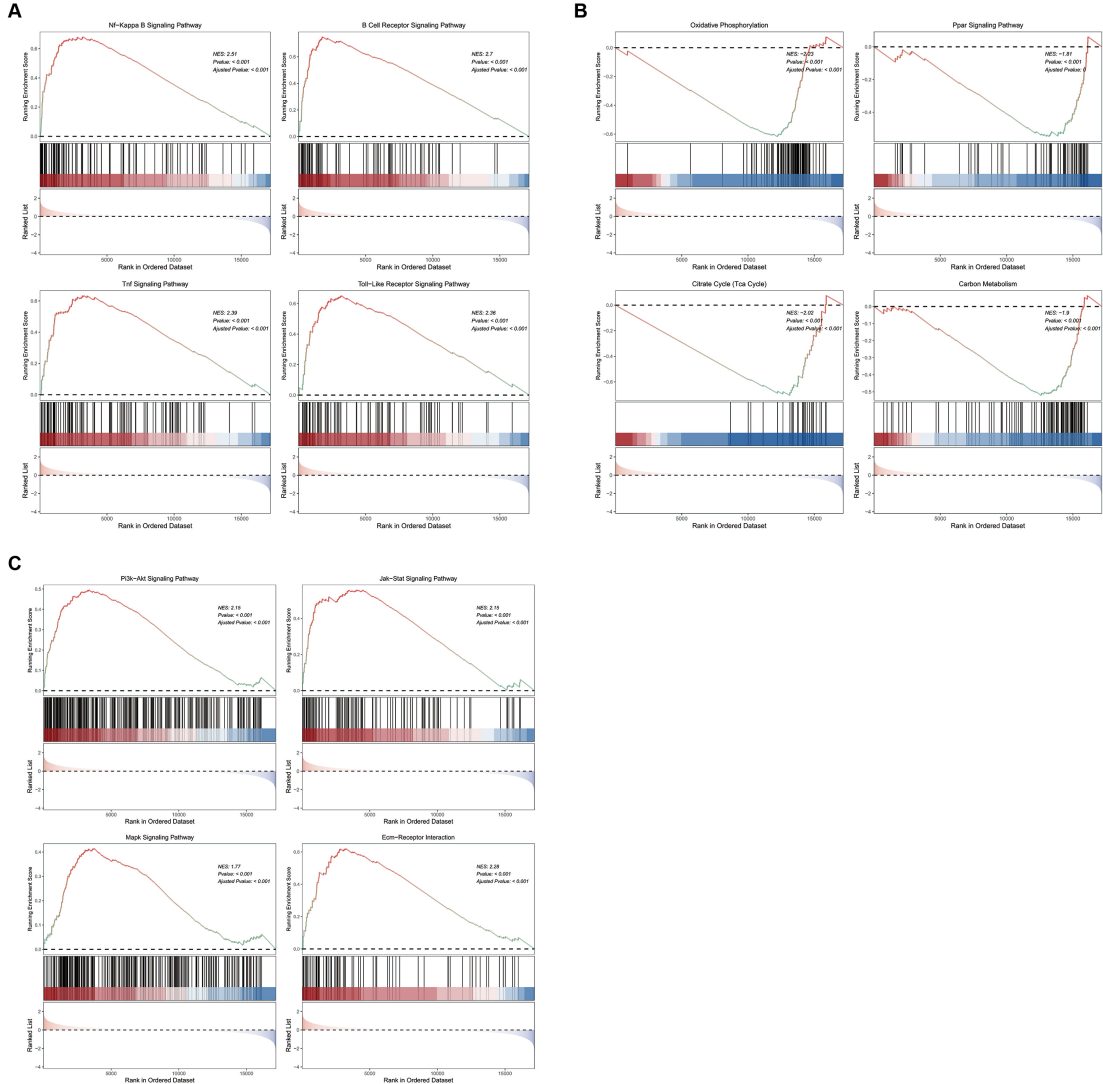
The GSEA enrichment pathways of differentially expressed genes. **(A)** Inflammation and immune signaling pathways activated by cluster 1 compared to cluster 2. **(B)** Metabolic related pathways inhibited by cluster 1 compared to cluster 2. **(C)** Insulin resistance related pathways activated by cluster 1 compared to cluster 2. The first part is the line graph of gene enrichment score. The horizontal axis represents each gene in the gene set, and the vertical axis represents the corresponding running enrichment score. The peak value of the line graph is the enrichment score, which is the NES value of the gene set. The genes before the peak value are the core genes in the gene set. The middle part of the second part marks the genes located under the gene set. The third part is a distribution map of rank values for all genes, with the vertical axis reflecting the relative expression levels of each gene in the gene set.

GSEA analysis on KEGG pathways demonstrated that vitamin D metabolic disturbance upregulated various inflammatory and immune signaling pathways, such as “Nf-Kappa B signaling pathway,” “B cell receptor signaling pathway,” “TNF signaling pathway,” and “Toll-like receptor signaling pathway” (Figure 5A). Simultaneously, it downregulated multiple metabolic pathways involved in maintaining glucose homeostasis and inhibiting inflammation, such as “PPAR signaling pathway,” as well as those participating in glucose metabolism, including “oxidative phosphorylation,” “citrate cycle (TCA cycle),” and “carbon metabolism” (Figure 5B). Furthermore, vitamin D metabolic disturbance upregulated several insulin resistance-related pathways, such as PI3K-AKT, JAK/STAT, MAPK, and ECM receptor-related signaling pathways (Figure 5C).

GSEA analysis on GO terms revealed that vitamin D metabolic disturbance upregulated immune components in CC (cellular component), such as “T cell receptor complex” and “immunological synapse,” while downregulating necessary components in sugar metabolism processes like “mitochondrial intermembrane space” and “mitochondrial tricarboxylic acid cycle enzyme” (Supplementary Figure S1C). In BP (biological process), it upregulated immune mechanisms like “adaptive immune response,” “B cell activation,” and “lymphocyte proliferation,” while simultaneously downregulating processes related to sugar metabolism, such as “cellular response to insulin stimulus” and “response to monosaccharide” (Supplementary Figure S1D). In MF (molecular function), there was an upregulation of factors related to chemotaxis and cytokine activity, such as “chemokine binding” and “cytokine activity,” while inhibiting activities related to transporter receptors, like “passive transmembrane transporter activity” and “transmembrane transporter activity” (Supplementary Figure S1E).

### 3.5 PPI network reveals dysregulation of immune system and inflammation induced by vitamin D metabolic disturbance

**Figure 6:**
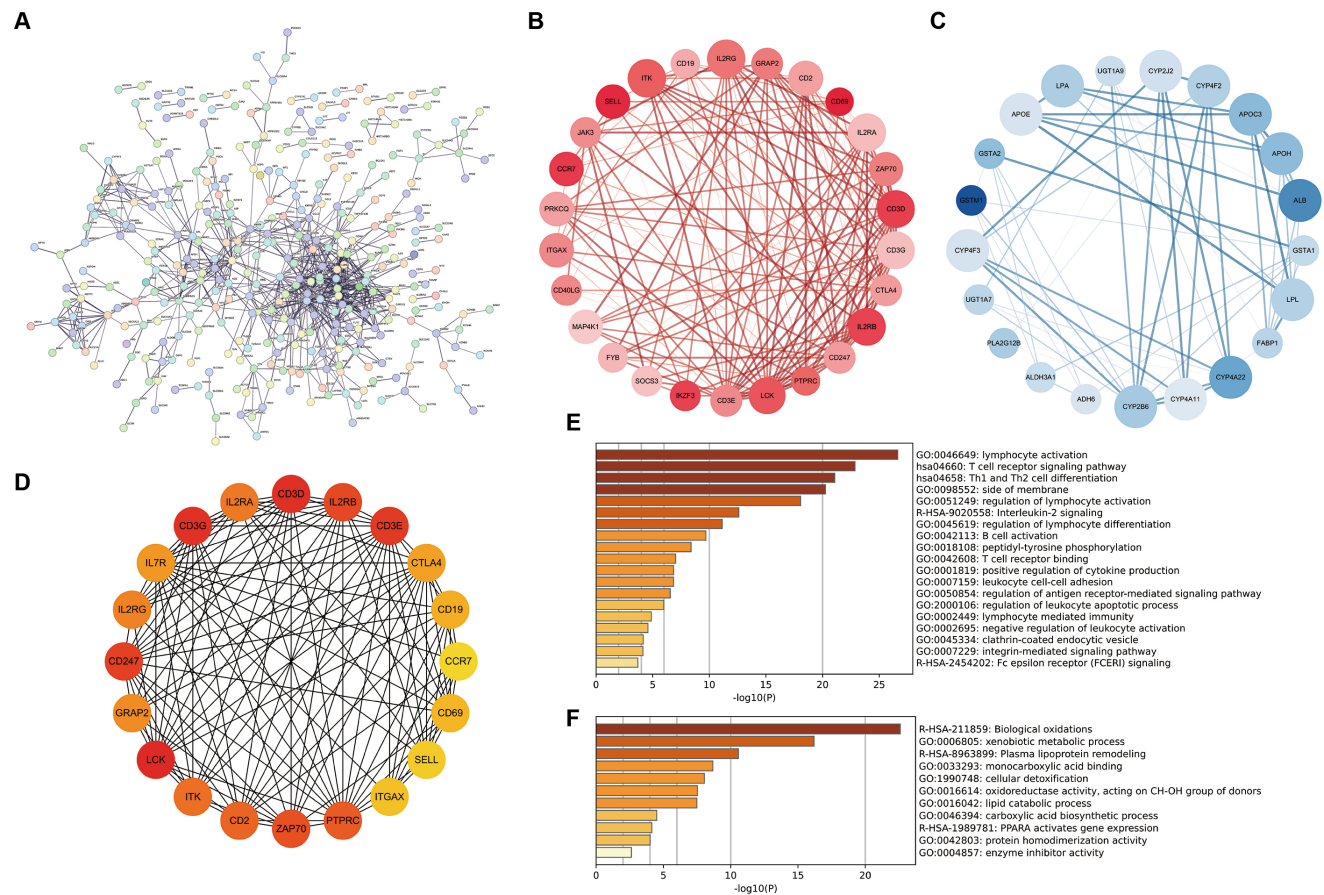
Construction and analysis of PPI network. **(A)** The PPI network of differentially expressed genes between cluster 1 and cluster 2, where each node represents a protein corresponding to a gene, and connections represent interactions between proteins. **(B,C)** The sub network with the highest and second scores calculated using the MCODE plugin. Red represents gene upregulation, blue represents gene downregulation. **(D)** Calculate the top 20 hub genes using the MCC algorithm in the CytoHubba plugin. **(E,F)** The enrichment pathway of genes in two MCODE sub networks. The horizontal axis is −log10 (*p*), and the vertical axis is the enriched pathway.

The DEGs of cluster 1 and cluster 2 construct the PPI network, and 18 MCODE sub-networks (Figure 6A). MCODE1 network primarily consisted of immune-inflammatory genes like CD69, CD3D, IL2RB, and CCR7, all upregulated in patients with vitamin D metabolic disturbance (Figure 6B). Enrichment analysis of this network indicated an upregulation of immune pathways, such as “lymphocyte activation” and “T cell receptor signaling pathway,” contributing to dysfunctional Th1 and Th2 cell functions (Figure 6E).

MCODE2 network, composed of CYB family genes and other metabolism-related genes, demonstrated downregulation of gene expression in patients with vitamin D metabolic disturbance (Figure 6C). Enrichment analysis of MCODE2 network highlighted the inhibition of pathways like “biological oxidations” and “PPARA activates gene expression,” suggesting suppression of oxidative metabolism pathways (Figure 6F). Hub genes identified by the CytoHubba plugin showed high similarity to genes in MCODE1 subnetwork (Figure 6D). These findings collectively indicate that vitamin D metabolic disturbance in DN patients predominantly activate immune and inflammatory pathways, leading to immune system imbalance and inflammation.

### 3.6 Immune cell activation in DN patients with vitamin D metabolic disturbance

**Figure 7:**
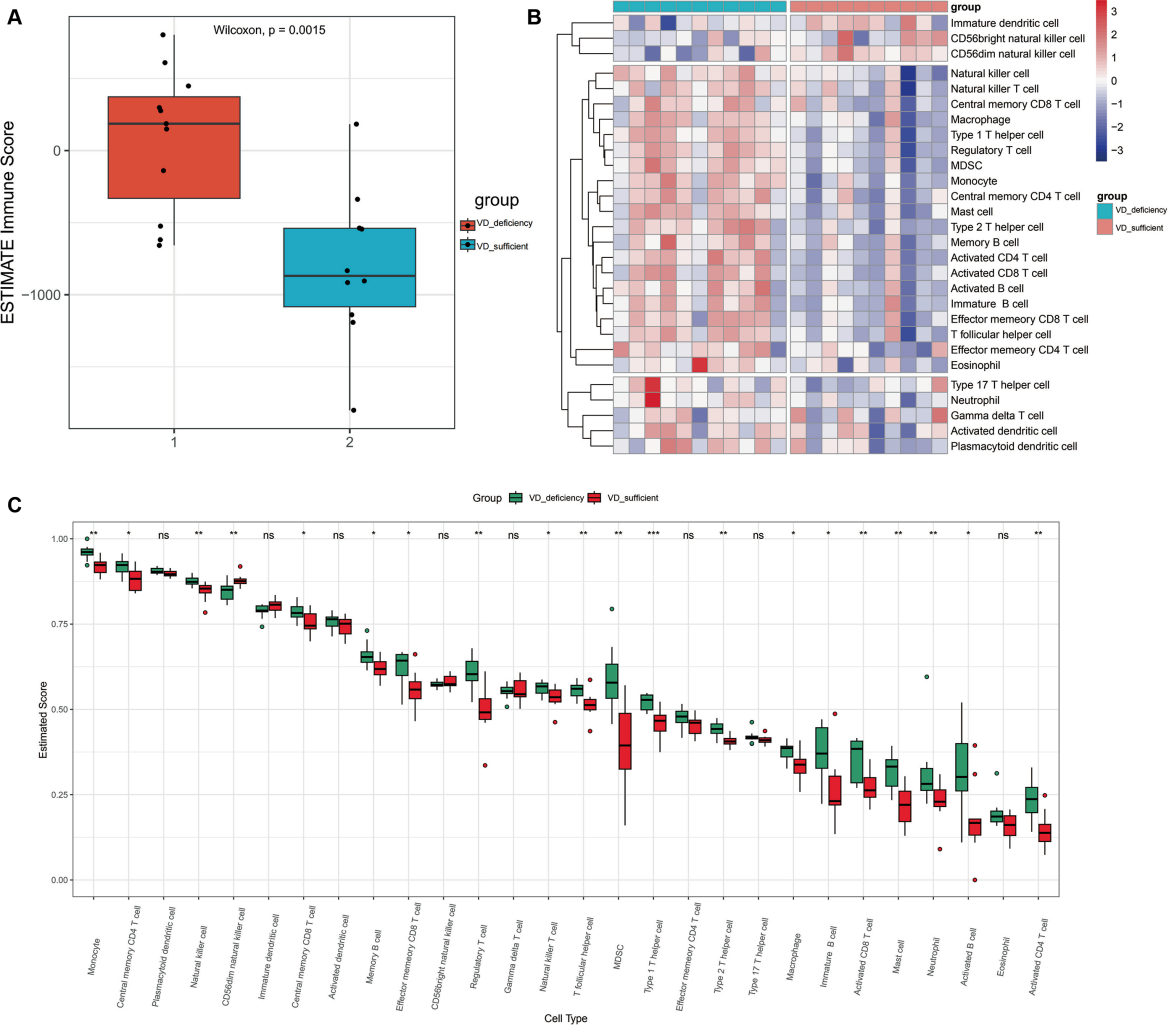
Immune infiltration analysis. **(A)** The immune scores for cluster 1and cluster 2 were calculated using the ESTIMATE algorithm. Immune score refers to the degree of infiltration of immune cells in tumor tissue, with larger values indicating higher levels of infiltration. **(B)** Heat maps of immune component scores calculated using ssGSEA. **(C)** Box plots of 28 immune related cell scores in patients with cluster 1 and cluster 2 calculated using the ssGSEA algorithm. *p* < 0.05 is considered statistically significant. ^ns^*p* > 0.05, ^*^p < 0.05, ^**^p < 0.01, and ^***^p < 0.001.

Both KEGG and PPI network analyses have revealed immune system imbalance and activation of various inflammatory pathways in patients with vitamin D metabolic disturbance in DN. This observation is further validated by immune infiltration analysis. ESTIMATE immune infiltration analysis demonstrates a significantly higher immune score in DN patients with vitamin D metabolic disturbance compared to those with relatively normal vitamin D metabolism (*p* = 0.0015), indicating abnormal activation of various immune functions in these patients (Figure 7A). Using the ssGSEA algorithm for infiltration analysis of 28 immune-related cell types, most immune cells show significantly higher expression in patients with vitamin D metabolic disturbance. Only a few, such as “CD56 bright natural killer cells,” exhibit a noticeable decrease in patients with vitamin D metabolic disturbance. This specific subset of NK cells, characterized by minimal differentiation in the blood, may play a distinctive role in DN (Figures 7B,C).

### 3.7 Construction of scRNA-seq Atlas for 15 cell types in DN patients

**Figure 8:**
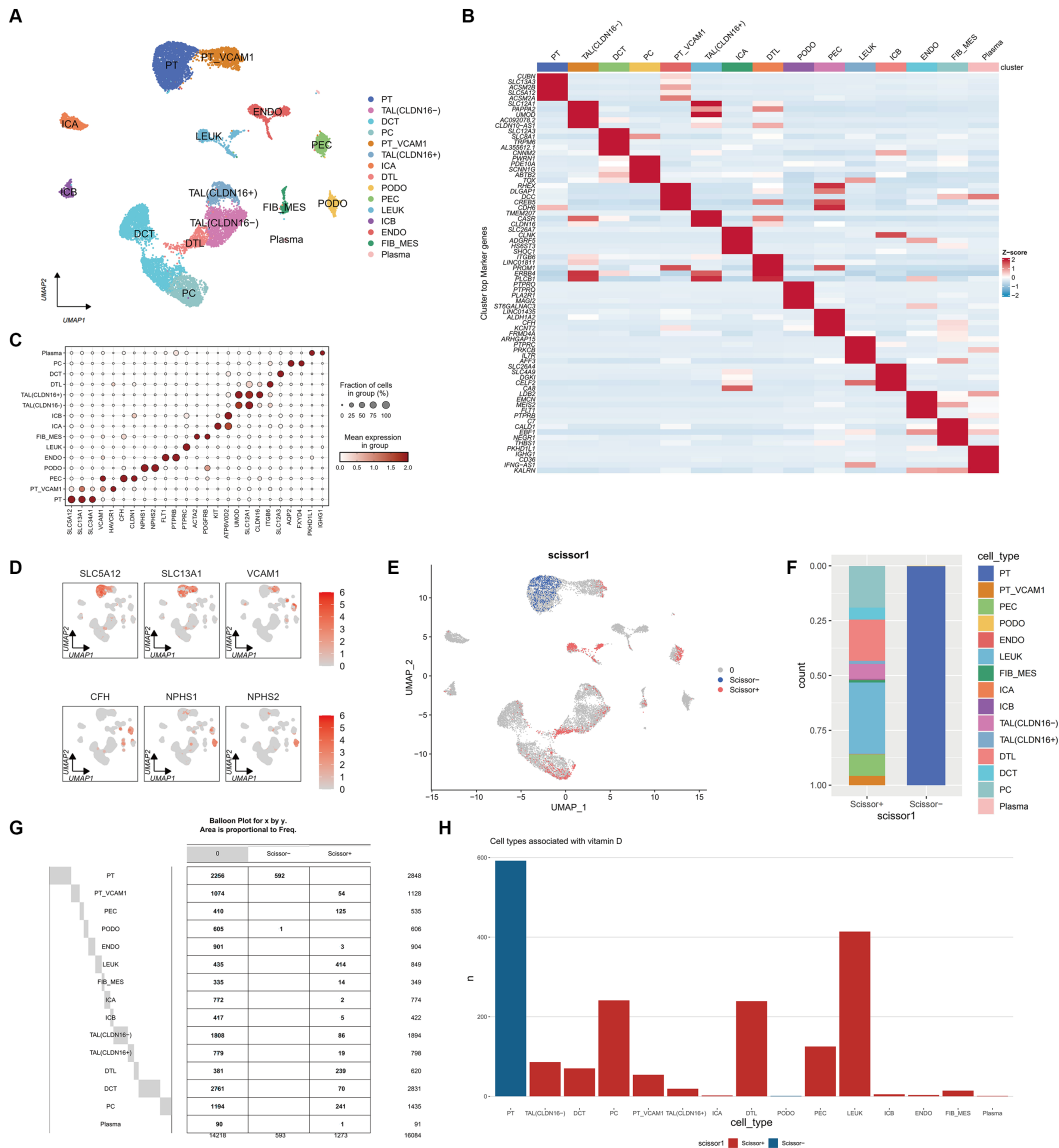
Single cell atlas of DN patients. **(A)** UMAP dimensionality reduction clustering diagram of 16,084 cells from DN patients, consisting of 15 cell types. PT, proximal tubule; PT VCAM1, VCAM1(+) proximal tubule; PEC, parietal epithelial cells; TAL CLDN16(−), thick ascending limb; TAL CLDN16(+), thick ascending limb; DCT, early distal convoluted tubule; PC, principal cells; ICA, type A intercalated cells; ICB, type B intercalated cells; PODO, podocytes; ENDO, endothelial cells; FIB-MES, fibroblasts and mesangial cells; LEUK, leukocytes; DTL, descending thin limb; plasma, plasma cells. **(B)** Specific genes of each cell subgroup compared to other cell subgroups. **(C)** A marker bubble chart for identifying cell types. The markers for each cell subpopulation are from the Cellmarker 2.0 database. **(D)** The expression of some marker genes on the UMAP map. **(E)** UMAP graph combining scRNA seq of DN patients with RNA seq of previous ADN patients using Scissor algorithm, where Scissor (+) represents a positive correlation with vitamin D metabolism disturbance phenotype and Scissor (−) represents a negative correlation. Zero represents cells that are not significantly correlated with vitamin D metabolism disturbance. **(F)** Stacked bar charts showing the proportion of different cell types in Scissor (+) and Scissor (−) cells. **(G)** Balloon plot of the number of cell subpopulations in Scissor (+) and Scissor (−) cells. **(H)** Bar charts showing the number of different cell types in Scissor (+) and Scissor (−) cells.

After integrating and filtering scRNA-seq data from 5 DN patients, a total of 16,084 cells were obtained. Comparative analysis with existing kidney tissue markers ultimately identified 15 distinct cell types (Figures 8A–D): PT, proximal tubule; PT VCAM1, VCAM1(+) proximal tubule; PEC, parietal epithelial cells; TAL CLDN16(−), thick ascending limb; TAL CLDN16(+), thick ascending limb; DCT, early distal convoluted tubule; PC, principal cells; ICA, type A intercalated cells; ICB, type B intercalated cells; PODO, podocytes; ENDO, endothelial cells; FIB-MES, fibroblasts and mesangial cells; LEUK, leukocytes; DTL, descending thin limb; plasma, plasma cells.

### 3.8 Scissor analysis reveals that vitamin D metabolism disturbance induce the aggregation of immune cells and are associated with renal injury

Using the Scissor algorithm, the analysis revealed that the combination of bulk RNA-seq and vitamin D phenotype with scRNA-seq data from 21 DN patients resulted in 1273 Scissor (+) cells positively correlated with vitamin D metabolism disturbance and 593 Scissor (−) cells negatively correlated (Figure 8E). Notably, vitamin D metabolism disturbance predominantly inhibited a subset of proximal tubule (PT), while the Scissor (+) cells promoted by vitamin D metabolism disturbance were distributed in PT VCAM1, PEC, LEUK, DTL, and PC (Figures 8F–H). Importantly, almost half of the cells in the LEUK subset showed a positive correlation with vitamin D metabolism disturbance, indicating that vitamin D metabolism disturbance promotes the aggregation of immune cells. Additionally, PT VCAM1 represents a distinct subset of PT cells with abnormal expression of VCAM1 and HAVCR1, which is a specific cell subset associated with kidney damage in DN patients. Vitamin D metabolism disturbance exerted opposite effects on two distinct subsets of PT and PT VCAM1, with only vitamin D metabolism-negative cells present in PT and only vitamin D metabolism-positive cells in PT VCAM1 (Figure 8G). This suggests that vitamin D metabolism disturbance may inhibit the function of normal PT cells while promoting the generation of abnormal PT cells, exacerbating kidney damage in DN patients.

### 3.9 Vitamin D metabolism disturbance are associated with various complications in DN patients

**Figure 9:**
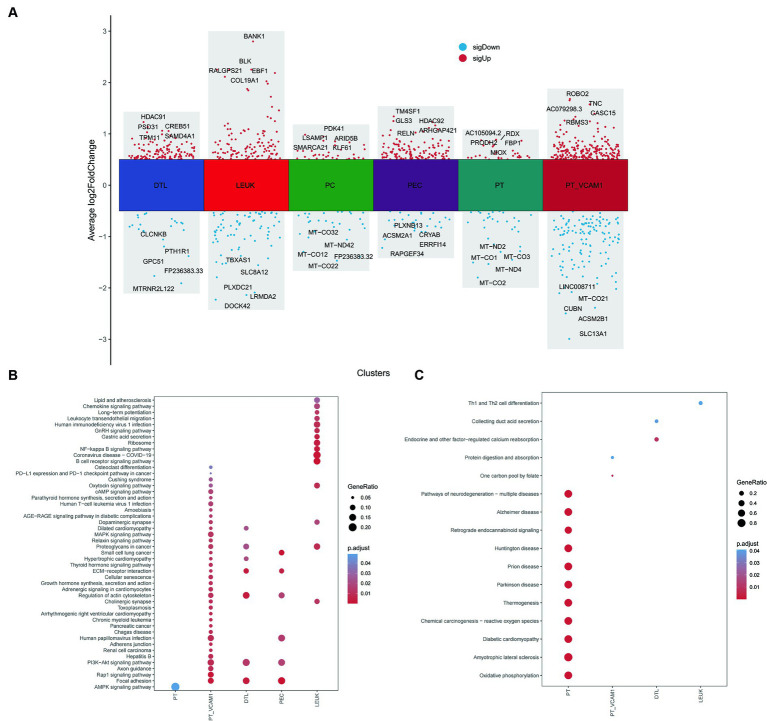
Differential and enrichment analysis between Scissor cells and normal cells. **(A)** Differential expression genes between Scissor cells and normal cells in PT, PT_VCAM1, PEC, PC, DTL and LEUK. **(B)** Common and different KEGG enrichment pathways enriched by upregulated genes of various cell types. **(C)** Common and different KEGG enrichment pathways enriched by downregulated genes of various cell types. The size of bubbles represents the proportion of enriched genes, and the color represents significance.

Further exploration of the differences between Scissor cells and corresponding normal cells in different renal tissue compartments related to vitamin D metabolism disturbance was conducted through differential enrichment analysis, as illustrated in Figure 9A. It was observed that Scissor-cells in the PT exhibit an upregulation of the AMPK signaling pathway compared to normal PT cells, suggesting that vitamin D metabolism disturbance downregulate the normal expression of this pathway in PT. In PT VCAM1, DTL, and PEC subsets, vitamin D metabolism disturbance-positive cells activate the PI3K-AKT signaling pathway and ECM receptor pathway, indicating an enhancement of insulin resistance in these segments (Figure 9B). Additionally, the “AGE-RAGE signaling pathway in diabetic complications” is significantly upregulated in PT VCAM1. In the LEUK cell subset, the positive portion of vitamin D metabolism disturbance significantly upregulates various immune and inflammatory pathways, including NF-κB pathways. Meanwhile, the downregulated genes in PT show enrichment in various diseases, such as “diabetic cardiomyopathy” and various neurological disturbance (Figure 9C), suggesting that vitamin D metabolism disturbance may induce neurologic disturbances and increase the risk of neurological complications in DN patients.

## 4 Discussion

DN is a slowly progressive disease and a leading cause of renal failure worldwide. Epidemiological studies indicate that 25 to 40% of patients with type 1 diabetes mellitus (T1DM) and 5 to 40% of those with T2DM ultimately develop DN (20). Previous research has suggested a potential protective role of vitamin D levels in the development of diabetes (21), but its specific role in DN remains to be investigated. Vitamin D is converted to 25-OH-D by hydroxylation in the liver, 25-OH-D includes 25-OH-D2 and 25-OH-D3, 25-OH-D3 is the main form of vitamin D in the body, and it is a common clinical indicator of vitamin D deficiency (VDD). In this study, the analysis of clinical data from normal, T2DM and DN patients showed that DN patients had lower levels of 25-OH-D3 compared to T2DM patients and normal subjects, and the proportion of patients with extreme vitamin D deficiency increased with the progression of DN, and the progression of diabetic nephropathy was significantly positively correlated with VDD. The kidney is the main metabolising organ of vitamin D. When the kidney is damaged by DN, not only vitamin D metabolism and activation will be disturbed to a certain extent, but also the glomerular filtration rate will be increased and tubular reabsorption is impaired, resulting in the loss of the protein-bound 25-OH-D3 due to proteinuria, and the level of 25-OH-D3 is negatively correlated with the UACR and positively correlated with the blood albumin, which is in agreement with our results. This is consistent with our results. It should be noted that there was a significant difference in age between T2DM, EDN and ADN (*p* < 0.05). The reason may be that DN belongs to a kind of chronic kidney disease, which gradually progresses from EDN to ADN with the prolongation of the disease, and the patients’ renal function continues to decline, resulting in a higher age percentage of ADN patients relative to T2DM and EDN.

Increasing evidence suggests that the activation of the vitamin D receptor (VDR) signaling pathway has various renal protective effects in DN patients, including anti-inflammatory, anti-proteinuria, anti-fibrotic, and protection against podocyte injury to maintain their survival (22, 23). Genetic trend analysis of 209 vitamin D DEGs obtained from public databases showed that some of the consistently down-regulated vitamin D DEGs were enriched in the “WP VITAMI D RECEPTOR PATHWAY” pathway. Recent studies have indicated that vitamin D/VDR can downregulate the expression of FOXO1 in diabetic patients, inhibiting iron-deficiency anemia in pancreatic β-cells (24). Furthermore, it is well known that cyp24A1 of the Cyp450 family is involved in the degradation of 25-OH-D-vitamins and is increased in damaged kidneys. However, the results of our analyses did not reveal differential expression of cyp24A1 during the progression of normal-EDN-ADN, which requires further attention.

As one of the characteristics of T2DM, immune activation and inflammation are highly correlated with an increased risk of diabetes (25). In vitamin D metabolism disturbance in DN patients, pathways such as “Nf-Kappa B signaling pathway,” “B cell receptor signaling pathway,” “TNF signaling pathway,” and “Toll-like receptor signaling pathway” are significantly upregulated. These pathways play crucial roles in immune responses (26–29). Immune infiltration analysis corresponds to these results, showing that the immune score of most immune cells in vitamin D metabolism disturbance patients is significantly higher than that in patients with relatively normal vitamin D metabolism, except for a few, such as “CD56 bright natural killer cell,” which is significantly reduced in vitamin D metabolism disturbance patients. CD56 bright natural killer cells are a small subset of CD56 NK cells that produce numerous cytokines to regulate the immune system (30). The role of this mechanism in DN is still unknown and requires further research. In recent decades, research on vitamin D has confirmed its important interactions with the immune system (31). The impact of vitamin D on the immune system in patients with DN is not yet clear. Our bioinformatics analysis showed that vitamin D metabolism disturbance is closely related to the abnormal regulation of the immune system, which can provide a new reference for immunotherapy in patients with DN.

Furthermore vitamin D metabolism disturbance down-regulate various metabolic pathways such as “PPAR signaling pathway,” “oxidative phosphorylation,” “citrate cycle (TCA cycle),” and “carbon metabolism.” Peroxisome proliferator-activated receptor gamma (PPARγ) is a ligand-activated nuclear receptor that regulates glucose and lipid metabolism (32). Insulin can enhance anti-inflammatory effects by upregulating the expression of PPAR-γ (33). Additionally, studies have shown that vitamin D can increase PPAR-γ coactivator 1α (PGC-1α) levels to reduce insulin resistance (34). Insulin resistance is a key factor leading to elevated blood sugar in patients with type 2 diabetes (35). We found abnormal upregulation of P13K-AKT, JAT/STAT, MAPK, and ECM receptor-related signaling pathways associated with insulin in vitamin D metabolic disturbance (36–38). Currently, only a small amount of basic and clinical research results support the beneficial effects of vitamin D in reducing insulin resistance (39, 40). However, the specific mechanisms remain unclear, and our analysis results can provide guidance in this direction.

scRNA-seq analysis validated the results of bulk RNA-seq, revealing that nearly half of the cells in the immune cell subset LEUK were positively correlated with vitamin D metabolism disturbance, and vitamin D metabolism disturbance positive cells significantly activated various immune and inflammatory pathways. Furthermore, it is intriguing that vitamin D metabolism disturbance is closely associated with the production of a specific cell subpopulation, PT VCAM1, that highly expresses the kidney injury factor HAVCR1. Previous studies have shown that HAVCR1 is one of the key factors in renal tubular injury, PT cell cycle arrest, and secondary glomerulosclerosis (41). Current studies suggest that vitamin D may be a potential therapeutic target for acute kidney injury (42), but the relationship between vitamin D and kidney injury in DN has not yet been reported. This discovery may provide clues for slowing the development of DN and rescuing kidney injury. Moreover, we found that the downregulated genes in vitamin D metabolism disturbance negative cells in PT were enriched in various neurological diseases, indicating that vitamin D metabolism disturbance may induce neurological disturbance, increasing the risk of nerve damage in DN patients, which is consistent with previous research results (43).

VDD or vitamin D metabolism disturbance is positively associated with the severity of renal injury. The mechanisms may involve abnormal regulation of the immune system by vitamin D metabolism disturbance, metabolic suppression, upregulation of insulin resistance and inflammatory signalling pathways. Further clinical and basic studies are needed to explore the interactions and molecular mechanisms of vitamin D and DN.

## Data availability statement

The original contributions presented in the study are included in the article/Supplementary material, further inquiries can be directed to the corresponding author.

## Ethics statement

This study has been reviewed and approved by Ethics Committee of Rocket Force Specialty Medical Center, Ethics No. KY2024002.Written informed consent has been obtained from individuals for the publication of any potentially identifiable images or data included in this article.

## Author contributions

JG: Writing – review & editing, Writing – original draft, Visualization, Formal analysis, Data curation, Conceptualization. XS: Writing – review & editing, Supervision. HO: Writing – review & editing, Supervision, Resources, Project administration. XC: Writing – review & editing, Supervision, Formal analysis. LZ: Writing – review & editing, Supervision, Formal analysis. CL: Writing – review & editing, Resources, Data curation. YD: Writing – original draft, Investigation. XW: Writing – review & editing, Resources, Project administration, Funding acquisition, Conceptualization.

## Footnotes

^1^ https://www.ncbi.nlm.nih.gov/geo/

^2^ https://www.string-db.org/

^3^ https://metascape.org/gp/
