# Supplementary material for: The association between vitamin D and the progression of diabetic nephropathy: insights into potential mechanisms
Source: Front Med (Lausanne). 2024 Jun 24;11:1388074. doi: 10.3389/fmed.2024.1388074 (PMC11228314; doi:10.3389/fmed.2024.1388074)
Supplement: Supplementary file 1 [file Data_Sheet_1.docx]

Supplementary Material

## 1.Supplementary Figures


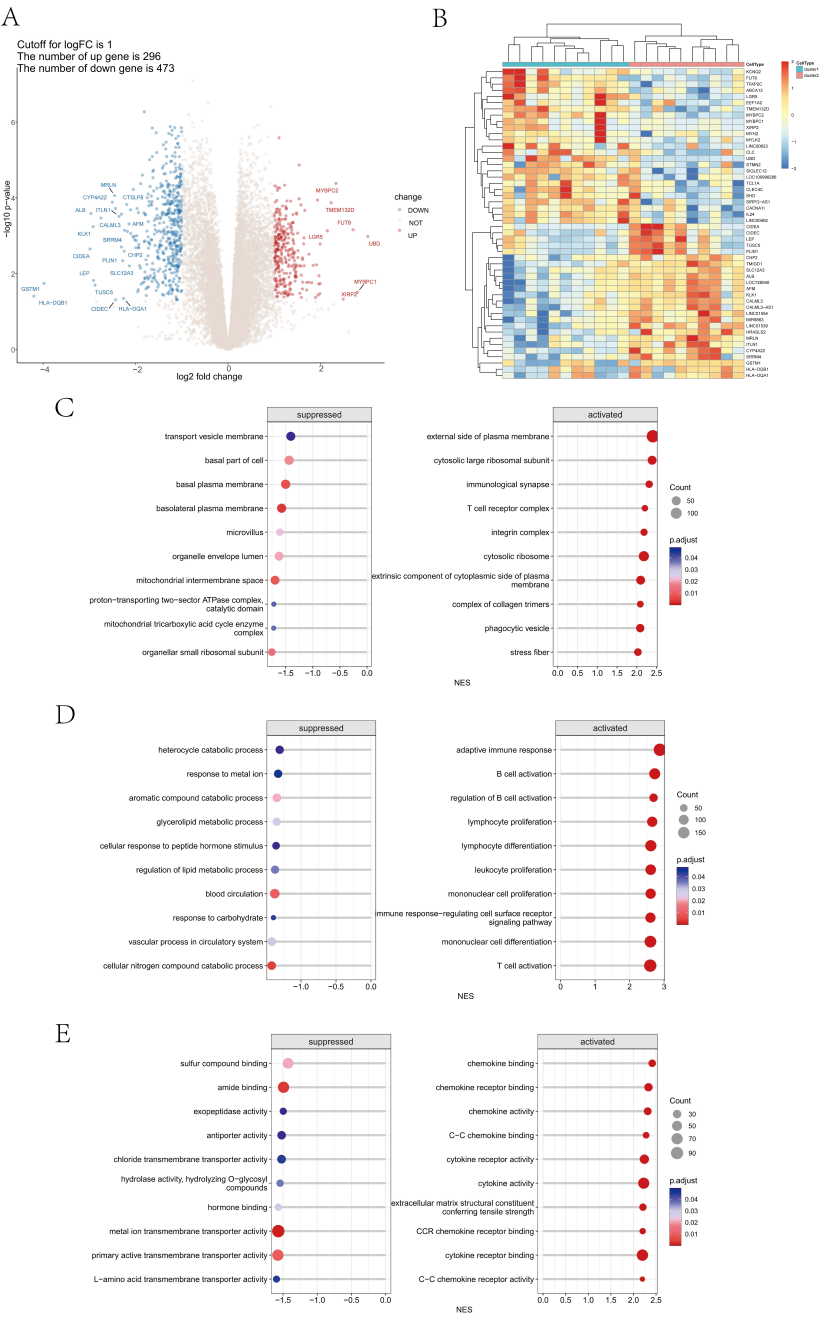


**Supplementary Figure 1.**Volcano map heat map and go analysis of differential genes. (A) Volcano map of differentially expressed genes between the cluster1 and cluster2 group. The horizontal axis is log 2 foldchange( foldchange is the multiple of gene expression differences ), and the vertical axis is - log10 p-value. (B) A differential gene expression heatmap between the cluster1 and cluster2 group, with the top 25 upregulated and top 25 downregulated genes selected based on the highest foldchange. (C-E) GO Pathways enriched in GSEA analysis. The horizontal axis NES represents enrichment score, positive numbers represent pathway activation in cluster1, and negative numbers represent pathway suppression.
